# Fat People

**Published:** 1872-05

**Authors:** 


### FAT PEOPLE.

A YOUNG medical student of twenty-two weighed three hundred and ten pounds, and not knowing whereunto these things might grow, he determined to abate the nuisance, and reasoned in this wise: Potash or lye water put into fat, makes soap, and water washes away soap; so I will take potash pills, and when it turns my fat to soap I will wash it away. So he drank potash water and swallowed potash pills; then he drank water to wash the fat out of him, and he scrubbed himself with water to wash the fat off of him; then he steamed himself in vapor baths to melt the fat out of him; next he had water splashed over him, then wound himself in wet sheets, and plastered himself with damp towels; altogether working harder than any galley slave. At the end of forty-one days he had got rid of thirty-four pounds of grease; feeling encouraged he persevered in the treatment forty days longer, when he rejoiced in the weight of two hundred and fifty-seven pounds, still measuring a yard and seven inches round about.

It is not unlikely that the three months' hard work diminished his weight, irrespective of other means.

A correspondent writes that she had weighed about two hundred and fifty pounds, and had reduced herself fifty pounds by Banting's system, without any observed ill results. Perhaps as safe and efficient a plan as any other is to live mainly on fruits, lean meat, drinking the least amount of any fluid, even water, and working hard in the open air every day.

